# Supplementary material for: Regulatory gene function handoff allows essential gene loss in mosquitoes
Source: Commun Biol. 2020 Sep 30;3:540. doi: 10.1038/s42003-020-01203-w (PMC7528073; doi:10.1038/s42003-020-01203-w)
Supplement: Supplementary file 1 — Supplementary Information [file 42003_2020_1203_MOESM1_ESM.pdf]

Supplemental Figures

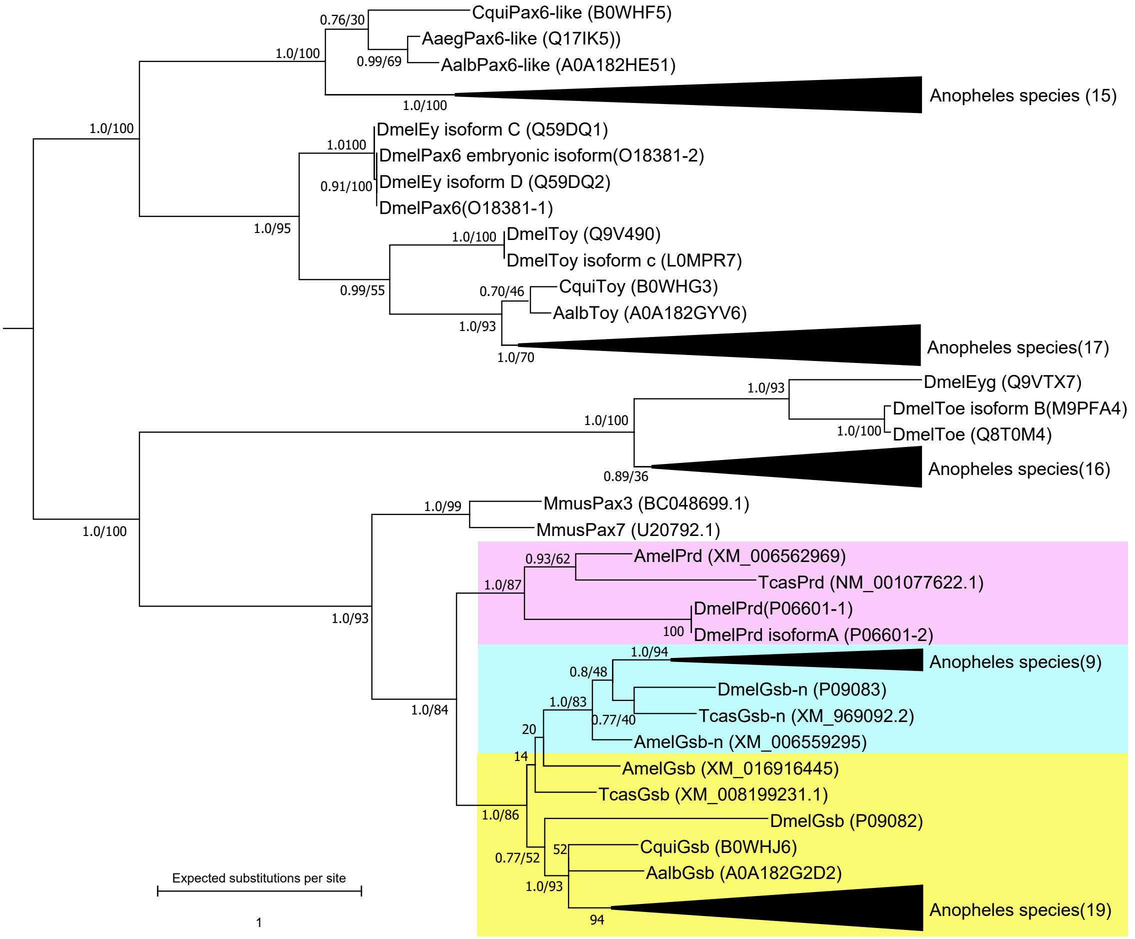

Supplementary Figure 1: A HMMER search of all publicly available mosquito genomes fails to isolate *paired* orthologs. Previously characterized *Pax* genes described in Figure 1 were aligned and used to build a hidden markov model to query mosquito and *Drosophila melanogaster* genomes in a HMMER search. Both the HMMER hits and previously characterized *Pax* genes were aligned and subjected to phylogenetic analysis to determine orthology. This analysis identified mosquito orthologs of Gsb (yellow), Gsb-n (blue), Eygone (Eyg)/Twin of Eygone (Toe) and Pax6/Eyless(Ey)/Toy (Twin of Eyeless). However, none of the mosquito hits cluster with known insect Prd sequences (pink). 19 published *Anopheles* genomes were included in the search. *Anopheles* hits all fell into several monophyletic clades which are shown condensed here. Branch length indicates expected substitutions per site (scale bar). Values indicate statistical support for a node when tree topology is determined by MrBayes (first value indicates posterior probability) or PhyML (second value indicates bootstrap support). Accession numbers and annotation IDs can be found in Supplementary Table 1.

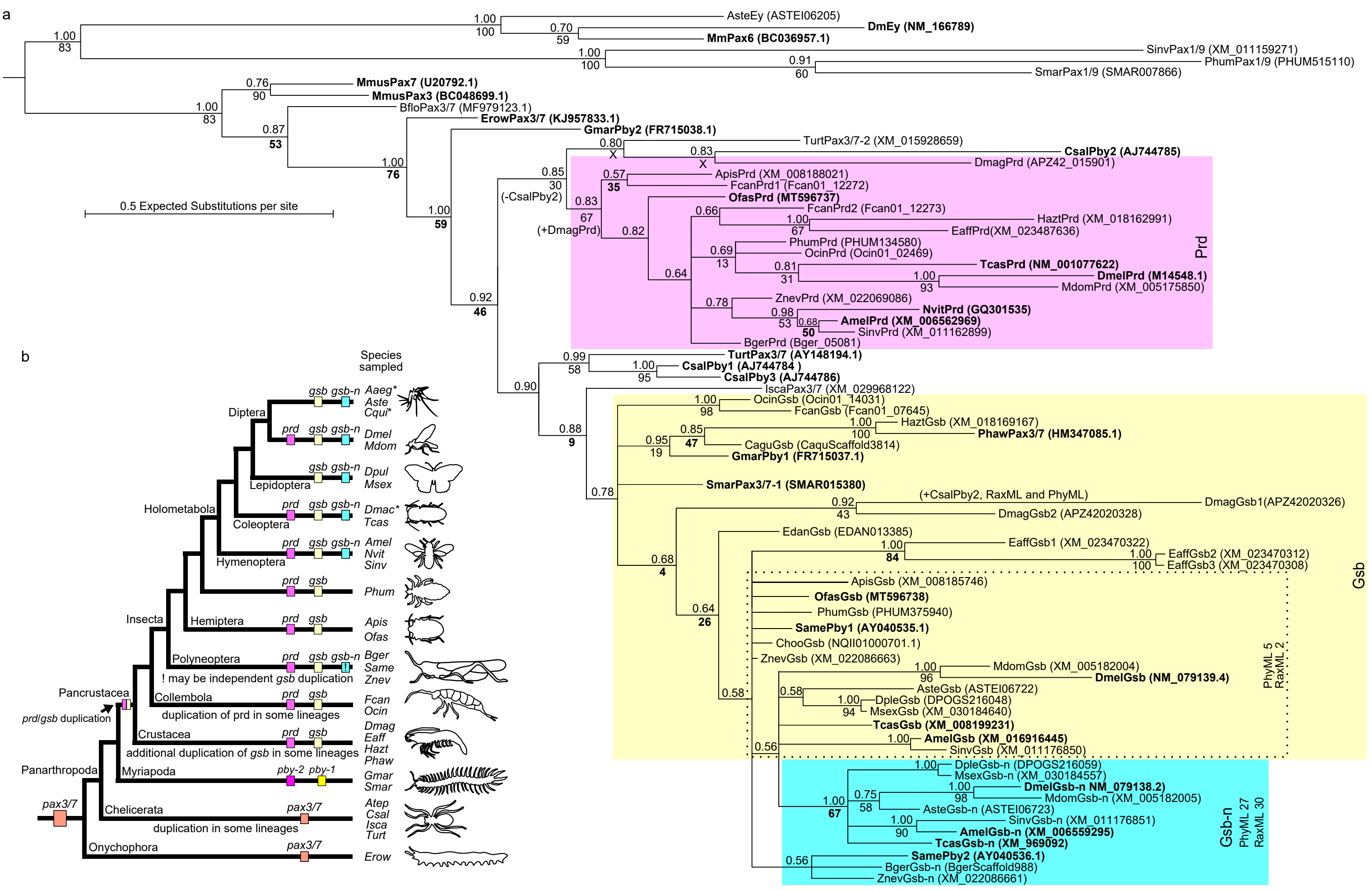

Supplementary Figure 2: *prd* and *gsb* Duplicated and Diverged at least by the emergence of Pancrustacea. a. Phylogenetic tree built from previously characterized *Pax* genes (names in bold) and arthropod genome BLAST hits. Depicted tree topology was determined by MrBayes. Values indicate statistical support for a node when tree topology is determined by MrBayes (posterior probability, above line), PhyML (bootstrap support, below line) or RaxML (bootstrap support, below line, bold). In some cases the clade is supported by another method only when one species is added to or removed from the clade (indicated in parentheses near support value). These analyses identify orthologs of Prd (pink) and Gsb (yellow) sequences in Pancrustacea lineages (crustaceans, non-insect hexapods, and insects). Gsb-n (blue) sequences are strongly supported as a clade in Holometabolous insects, with weaker support suggesting a possible common origin with Polyneoptera sequences. Insect Gsb sequences loosely form a clade when ML methods are used to determine topology (dotted box). b. Cartoon schematic depicting *Pax3/7* gene collections in different arthropod lineages. Duplication of *Pax3/7* to generate *prd* and *gsb* is indicated at the base of Pancrustacea. Sampled species with * indicate data incorporated from Supplementary Figure 4. ! indicates that "*gsb-n*" in Polyneoptera may instead represent an independent duplication of *gsb* rather than true orthology. Note that *Smar-pby2* was identified by Green and Akam (2013), but omitted from this analysis due to short alignment length[1]. Tree topology based on Misof et al., 2014[2].

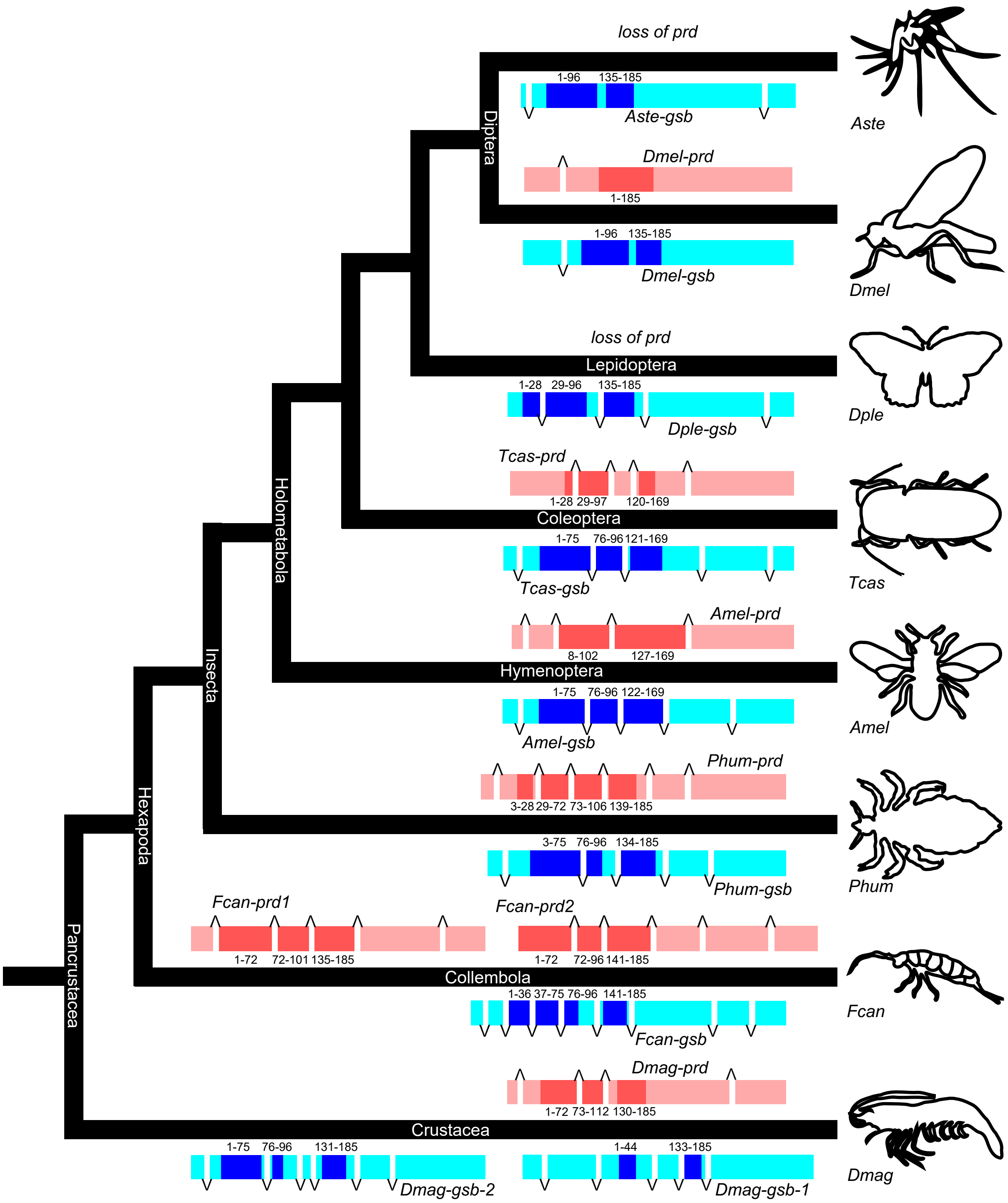

Supplementary Figure 3: *prd* and *gsb* gene structures from representative Pancrustacea lineages. Gene structures of *gsb* (blue) and *prd* (pink) from indicated species were copied from Ensembl Metazoa release 47 and assessed for conservation. Alignment to BLAST query (*Dmel-prd* AA 33-218), a highly conserved sequence containing a partial paired domain followed by a partial homeodomain, is indicated by red (*prd*) or dark blue (*gsb*) bars within the gene structure. Numbers indicate which amino acids of the 185 AA long query are represented by the aligned region. Introns are marked by V's and are not drawn to scale. All assessed *prd* and *gsb* orthologs contain a minimum of one intron, but most have several. Both *prd* and *gsb* have the paired (approx. the first 100 AA) and homeodomain (approx. the last 30 AA) sequences separated into different exons. *prd* sequences frequently have an intron inserted at position 72 of the query sequence while gsb sequences have an intron at position 75. Consolidation of sequences and loss of introns appears to be a derived feature of Diptera. The commonalities are suggestive of an origin by tandem duplication as opposed to retrotransposon.

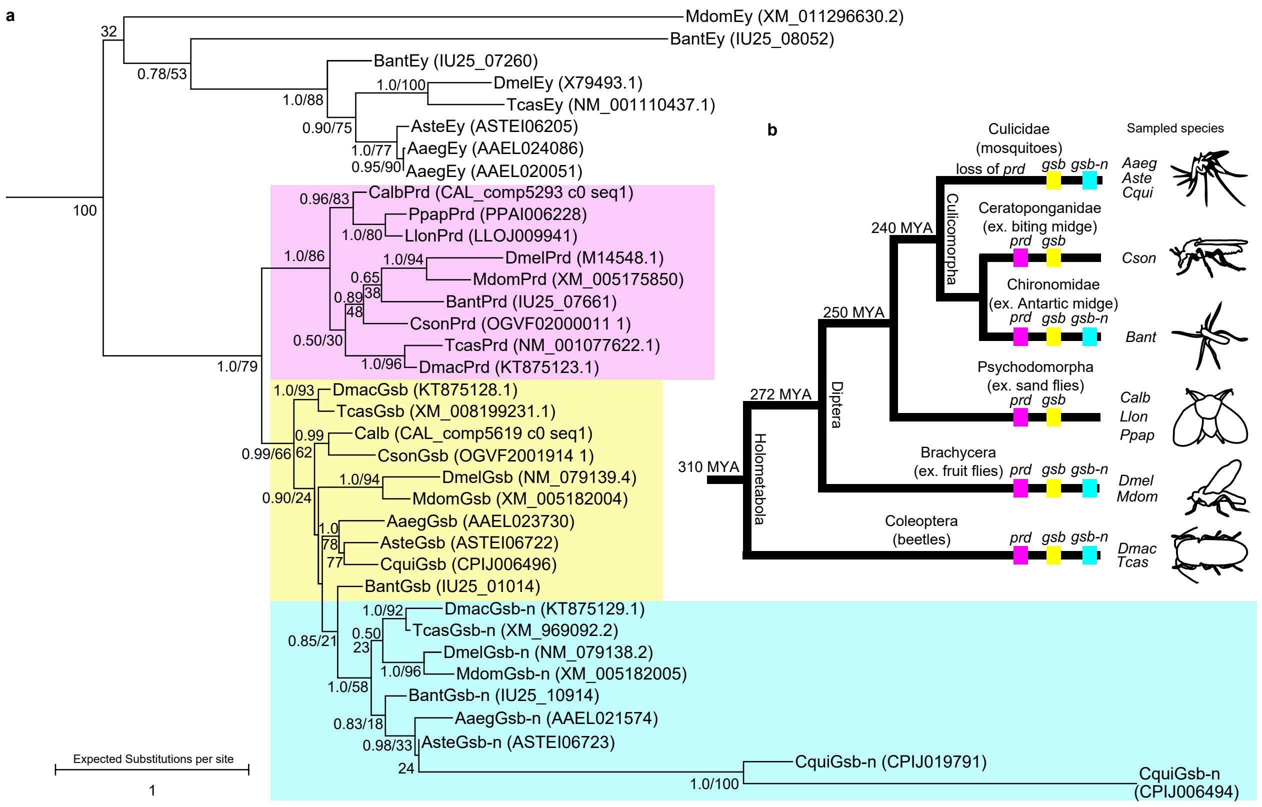

Supplementary Figure 4: Prd is present in the genomes of the closest relatives of mosquitoes, but not in mosquitoes. a A phylogenetic tree built from previously characterized *Pax* genes and dipteran genome and transcriptome BLAST hits identifies orthologs of Prd (pink), Gsb (yellow), Gsb-n (blue), and Pax6. None of the mosquito hits cluster with known insect Prd sequences (pink), yet these genes are present in multiple other dipteran lineages including midges (*i.e. Bant*, *Cson*), which represent a sister superfamily (Chironomoidea) within the same infraorder as mosquitoes (Culicomorpha). Branch length indicates expected substitutions per site (scale bar). Values indicate statistical support for a node when tree topology is determined by MrBayes (first value indicates posterior probability) or PhyML (second value indicates bootstrap support). Accession numbers and annotation IDs can be found in Supplementary Table 1. b Cartoon schematic depicting *Pax3/7* gene collections in different dipteran lineages. Loss of *prd* is specific to mosquitoes among its Nematoceran fly relatives. Tree topology based on Wiegmann et al., 2011[3].

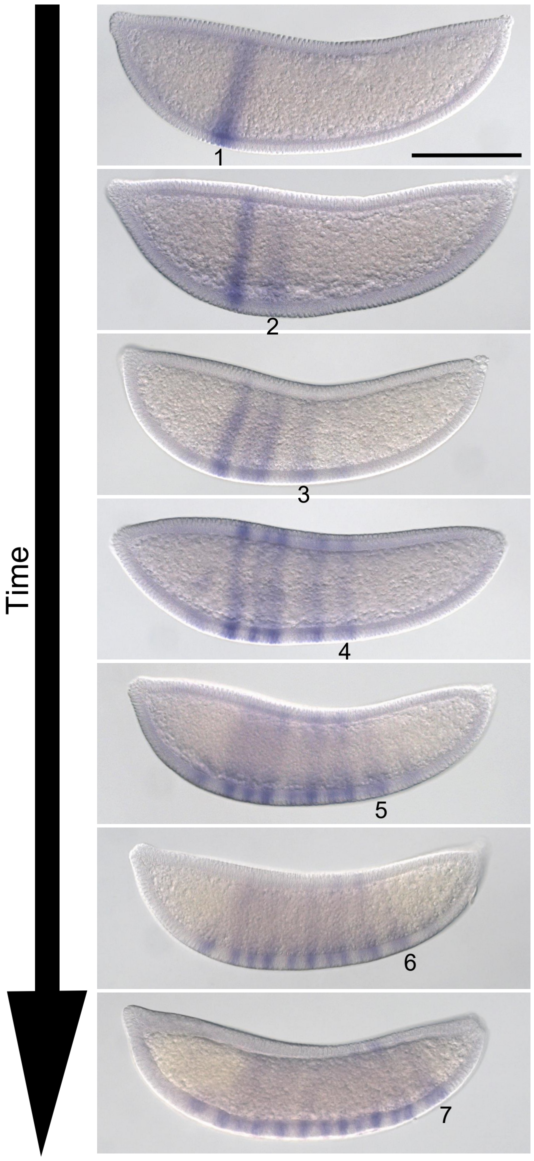

Supplementary Figure 5: *gsb* pair-rule stripes arise sequentially in *Anopheles* embryos. I*n situ* hybridization of *Anopheles* embryos throughout the blastoderm stage reveals broad stripes that arise one at a time and split into two before the appearance of the next pair-rule-stripe. There are seven total *Aste*-pair-rule stripes (numbered), as in *Drosophila*. Embryo stages are in order of increasing time with the youngest at the top and the oldest at the bottom, and in a lateral view with the anterior on the left. Scale bar indicates 100 µm.

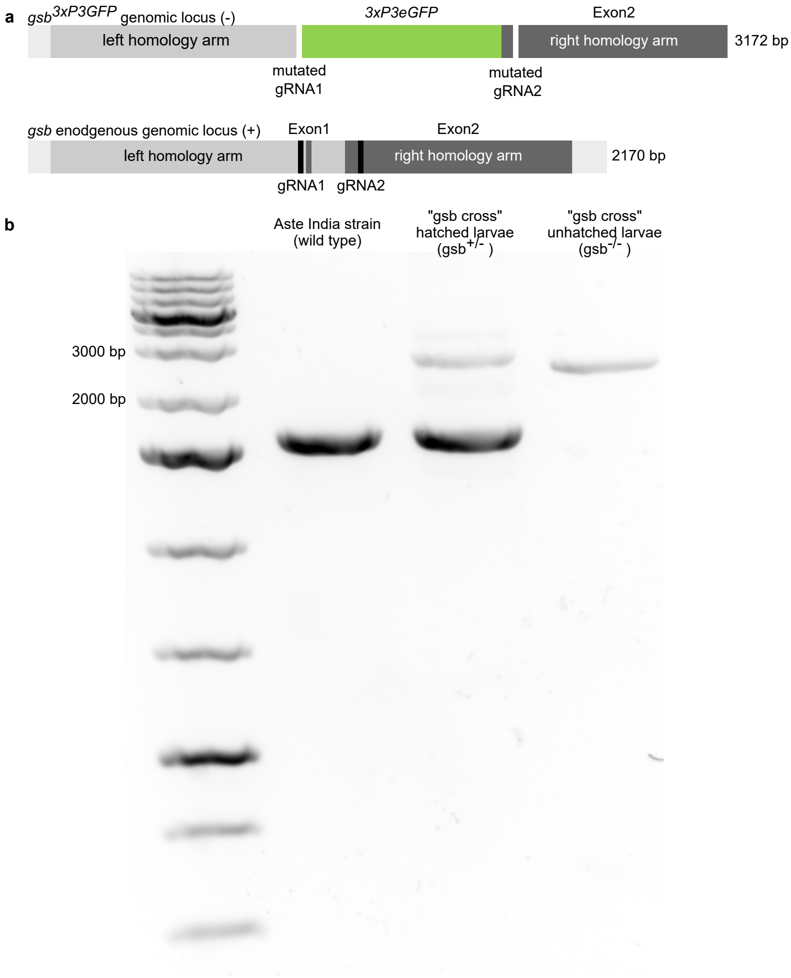

c *>Astegsb*^{3xP3GFP}

AGTGAAAGGNGAAATCGGACCACGGATGCCGNGGATTGATGTCNTNACTGTTTCCCCCCATCGGAGCAG
TCGGGTACGCTCTTAATAAGCGTGCAACCTATGA**AAAGGTGTCCGCAACTTCAGG**GATGGGGATTGGGG
ATTTGTTTCAACCCTTGCAACAGTTTTGTTGCTTCCTTGTTTTAATTTCNGAAACATGTGTTTTCGGGGTGG
AATTCTAAGCAAAACAGGAGCCTAATGGTCCTTCCTTCCGGATGCATATTTTTACACTCTACAACGCTGCC
CTGTGCGTTGTGTTGCGTGTGCTTGAGTTTGATTGCAATTTACGCCTAATTGATGACACTGATTAGCATGT
ACAGTGCGTCTGCGCTTGTGACCACAACATGCTTCGGTTTTTATTGCCCAGTCGTTAAACCGGTTACGGA
GAGGGTGGTGAAAACAACGGAAGAACGAAGGCGAAAGCATATCATTCAACTTTTCATCGACATGATTGAG
TTGATTAACGGCAAAGGCATGGAAGACATGAAGACAAGCAAAAAGGCAATTTGTTTTTGAATTTCCTACCT
TGTGGAACGATTTAGGCGTTCGTCCCTCCAACGGAGAATGATAAAGAAATCGTTTTCCAGCCCACCGGAC
CGGAAGCATCTCGTGTCGAACGTGTCAGAGCACGGATCGCCCCGAACCGAACCGGCGGAAGTCTTTTAA
TTGATTTAAAGTTCTCACCCAATCACCTGACTGGCTTGTAATCCGACCGGTGGCGGTTGGGCTGGGAACT
TCTCAATCAAAGACCGGCGAGGAAGTCAAAGAGGCAAAAGGGCCACTAATGTGACCGAaCAATGACACC
NTCGNNNNNGCCTTTCAGCAAACCTGCAACACGAGCCCTAGTCCTACTCCTCCNTCGCACTACGGCGGT
ACTGTGGGACGATCGCTTTGATCTTGATCTTGTGAGGATCATCGCCACCGTAGGTCCCTAGCGTGGAACG
CACTGCATCGCCGAGTGTGGTTCTGGGTTGTGGTTCCCACAAATGGTTAATTCGAGCTCGCCCGGGGAT
CTAATTCAATTAGAGACTAATTCAATTAGAGCTAATTCAATTAGGATCCAAGCTTATCGATTTCGAACCCTC
GACCGCCGGAGTATAAATAGAGGCGCTTCGTCTACGGAGCGACAATTCAATTCAAACAAGCAAAGTGAAC
ACGTCGCTAAGCGAAAGCTAAGCAAATAAACAAGCGCAGCTGAACAAGCTAAACAATCGGGGTACCGCTA
GAGTCGACGGTACCGCGGGCCCGGGATCCACCGGTCGCCACCATGGTGAGCAAGGGCGAGGAGCTGTT
CACCGGGGTGGTGCCCATCCTGGTCGAGCTGGACGGCGACGTAAACGGCCACAAGTTCAGCGTGTCCG
GCGAGGGCGAGGGCGATGCCACCTACGGCAAGCTGACCCTGAAGTTCATCTGCACCACCGGCAAGCTG
CCCGTGCCCTGGCCCACCCTCGTGACCACCCTGACCTACGGCGTGCAGTGCTTCAGCCGCTACCCCGA
CCACATGAAGCAGCACGACTTCTTCAAGTCCGCCATGCCCGAAGGCTACGTCCAGGAGCGCACCATCTT
CTTCAAGGACGACGGCAACTACAAGACCCGCGCCGAGGTGAAGTTCGAGGGCGACACCCTGGTGAACC
GCATCGAGCTGAAGGGCATCGACTTCAAGGAGGACGGCAACATCCTGGGGCACAAGCTGGAGTACAACT
ACAACAGCCACAACGTCTATATCATGGCCGACAAGCAGAAGAACGGCATCAAGGTGAACTTCAAGATCCG
CCACAACATCGAGGACGGCAGCGTGCAGCTCGCCGACCACTACCAGCAGAACACCCCCATCGGCGACG
GCCCCGTGCTGCTGCCCGACAACCACTACCTGAGCACCCAGTCCGCCCTGAGCAAAGACCCCAACGAGA
AGCGCGATCACATGGTCCTGCTGGAGTTCGTGACCGCCGCCGGGATCACTCTCGGCATGGACGAGCTGT
ACAAGTAAAGCGGCCGCGACTCTAGATCATAATCAGCCATACCACATTTGTAGAGGTTTTACTTGCTTTAA
AAAACCTCCCACACCTCCCCCTGAACCTGAAACATAAAATGAATGCAATTGTTGTTGTTAACTTGTTTATTG
CAGCTTATAATGGTTACAAATAAAGCAATAGCATCACAAATTTCACAAATAAAGCATTTTTTTCACTGCATT
CTAGTTGTGGTTTGTCCAAACTCATCAATGTATCTTAGAGATGGCGTCCTCCGGAGTGCGGCCCTGCGTT
ATCTCACGCCAGCTCCGAGTATCGCACGGTTGCGTATCGAAAATTCTGAACCGATACCAGGAAACGGGCT
CCATCCGCCCGGGAGTCATCGGAGGCTCGAAACCGCGTGTGTCCACCCCAGAAATTGAGGCTCGCATCG
AGGAACTGCGCAAAGCGAACCCGGGCATCTTCAGCTGGGAAATCCGGGACAAGCTCATTAAGGATGGGC
TGTGTGACAAAACATCTGCCCCGTCCGTCAGCTCCATCAGCCGGCTACTGCGCGGTGGTCGTCGCGATG
ATTTGGATATGCGTGGCAATCATTCGATCAACGGCATCCTGGGAGGATCGTCTTGTGACGAGGACAGCGA
TACCGAATCCGAGCCAGGAATTACGCTGAAGCGCAAGCAACGCCGATCCAGGACAACGTTCAACGGTGA
ACAGCTCGAGGCGCTGGAAATTGCATTCAGCCGTACCCAGTACCCAGATGTTTACACGCGCGAAGAGCT
CGCCCAGAAGACAAAGCTTACCGAGGCACGCGTGCAGGTGTGGTTCTCCAACCGTCGGGCCCGTCTGC
GCAAGCACATGAGCTCCCAGCAAATGGTGGCCTTCGGTGCCGCACAGTATCCTTCACAATTCGAACAACA
GGCCGTTGCCGTTGCCGCCGTTACAGCATCGTCCGGTTCCGGTTCGGGATCGCCCATGGGAGCCGGTG
GCTGGTCCCAGTGCTACTCTTCCTCCCCGAACCAAACC**GCTTCGATTCATGCACCGTC**ACAGTCCTCTTA
CAATCCGATGGCCCAAACCATGATCCATCCGA

Supplementary Figure 6: Larvae with pair-rule defects are homozygous for *gsb*^{3xP3GFP} allele. a A schematic of CRISPR- engineered *gsb*^{3xP3GFP} and wild type *gsb* alleles. b Gel of PCR amplification products of *gsb* alleles from wild type, heterozygous, and pair-rule mutant larvae genomic DNA. Genomic DNA from pair-rule larvae only produces the larger band, indicative of *3xP3GFP* insertion. This validates the inference that pair-rule larvae are *gsb* homozygous mutants. Bands appear smaller than actual size due to presence of PCR reaction buffer in sample. c Sanger sequencing of 3 kb band from b confirms *3xP3GFP* (green sequence) and flanking homology arms (light and dark grey) have inserted into the correct genomic location. Primers used to amplify 5' and 3'-most ends of the homology arms are bold. Text with no highlighting corresponds to the genomic regions immediately outside of the homology arms, and match the appropriate locations upstream and downstream of the gsb start site. Primers outside of homology arms used to amplify and sequence insertion are listed in Supplementary Table 5.

Supplementary Tables

Supplemental Table 1

Species	Species abbreviation	Gene name	Gene abbreviation	Accession number or annotation ID	source	Alternate accession or annotation ID	Alternate source
Aedes aegypti	Aaeg	gooseberry	gsb	AAEL023730	Vectorbase		
Aedes aegypti	Aaeg	gooseberry-neuro	gsb-n	AAEL021574	Vectorbase		
Aedes aegypti	Aaeg	eyeless/paired box 6	ey/pax6	AAEL024086	Vectorbase		
Aedes aegypti	Aaeg	eyeless/paired box 6	ey/pax6	AAEL020051	Vectorbase	Q17IK5	EMBL-EBI Reference proteomes
Aedes albopictus	Aalb	gooseberry	gsb	A0A182G2D2	EMBL-EBI Reference proteomes		
Aedes albopictus	Aalb	paired box 6-like	pax6-like	A0A182HE51	EMBL-EBI Reference proteomes		
Aedes albopictus	Aalb	twin of eyeless	toy	A0A182GYV6	EMBL-EBI Reference proteomes		
Anopheles albimanus	Aalbi	eyegone	eyg	A0A182FUV8_ANOAL	EMBL-EBI reference proteomes		
Anopheles albimanus	Aalbi	gooseberry	gsb	A0A182F457_ANOAL	EMBL-EBI reference proteomes		
Anopheles albimanus	Aalbi	eyeless/paired box 6	ey/pax6	A0A182FJE8_ANOAL	EMBL-EBI reference proteomes		
Anopheles albimanus	Aalbi	paired box 6-like	pax6-like	A0A182FIK9_ANOAL	EMBL-EBI reference proteomes		
Anopheles arabiensis	Aara	eyegone	eyg	A0A182HNP4_ANOAR	EMBL-EBI reference proteomes		
Anopheles arabiensis	Aara	gooseberry	gsb	A0A182HX18_ANOAR	EMBL-EBI reference proteomes		
Anopheles arabiensis	Aara	gooseberry-neuro	gsb-n	A0A182HX19_ANOAR	EMBL-EBI reference proteomes		
Anopheles arabiensis	Aara	eyeless/paired box 6	ey/pax6	A0A182HIP9_ANOAR	EMBL-EBI reference proteomes		
Anopheles arabiensis	Aara	paired box 6-like	pax6-like	A0A182HIQ4_ANOAR	EMBL-EBI reference proteomes		
Anopheles atroparvus	Aatr	eyegone	eyg	A0A182JJ47_9DIPT	EMBL-EBI reference proteomes		
Anopheles atroparvus	Aatr	gooseberry	gsb	A0A182IQH2_9DIPT	EMBL-EBI reference proteomes		
Anopheles atroparvus	Aatr	gooseberry-neuro	gsb-n	A0A182J2X2_9DIPT	EMBL-EBI reference proteomes		
Anopheles atroparvus	Aatr	eyeless/paired box 6	ey/pax6	A0A182JAU5_9DIPT	EMBL-EBI reference proteomes		
Anopheles christyi	Achr	eyegone	eyg	A0A182KB38_9DIPT	EMBL-EBI reference proteomes		
Anopheles christyi	Achr	gooseberry	gsb	A0A182JXS4_9DIPT	EMBL-EBI reference proteomes		
Anopheles christyi	Achr	eyeless/paired box 6	ey/pax6	A0A182KGF7_9DIPT	EMBL-EBI reference proteomes		
Anopheles christyi	Achr	paired box 6-like	pax6-like	A0A182KDT3_9DIPT	EMBL-EBI reference proteomes		
Anopheles coluzzii	Acol	gooseberry	gsb	A0A182LN36_9DIPT	EMBL-EBI reference proteomes		
Anopheles coluzzii	Acol	gooseberry-neuro	gsb-n	A0A182LN35_9DIPT	EMBL-EBI reference proteomes		
Anopheles culicifacies	Acul	eyegone	eyg	A0A182MEE3_9DIPT	EMBL-EBI reference proteomes		
Anopheles culicifacies	Acul	gooseberry	gsb	A0A182LXT3_9DIPT	EMBL-EBI reference proteomes		
Anopheles culicifacies	Acul	eyeless/paired box 6	ey/pax6	A0A182MEA2_9DIPT	EMBL-EBI reference proteomes		
Anopheles culicifacies	Acul	paired box 6-like	pax6-like	A0A182MLL5_9DIPT	EMBL-EBI reference proteomes		
Anopheles darlingi	Adar	gooseberry	gsb	W5JIC1_ANODA	EMBL-EBI reference proteomes		
Anopheles darlingi	Adar	gooseberry-neuro	gsb-n	W5JBE7_ANODA	EMBL-EBI reference proteomes		
Anopheles darlingi	Adar	eyeless/paired box 6	ey/pax6	W5JQF9_ANODA	EMBL-EBI reference proteomes		
Anopheles darlingi	Adar	paired box 6-like	pax6-like	W5JPV4_ANODA	EMBL-EBI reference proteomes		
Anopheles dirus	Adir	eyegone	eyg	A0A182NPM1_9DIPT	EMBL-EBI reference proteomes		
Anopheles dirus	Adir	gooseberry	gsb	A0A182NQ09_9DIPT	EMBL-EBI reference proteomes		
Anopheles dirus	Adir	Paired box 6	ey/pax6	A0A182N018_9DIPT	EMBL-EBI reference proteomes		
Anopheles dirus	Adir	paired box 6-like	pax6-like	A0A182N013_9DIPT	EMBL-EBI reference proteomes		
Anopheles epiroticus	Aepi	eyegone	eyg	A0A182PL71_9DIPT	EMBL-EBI reference proteomes		
Anopheles epiroticus	Aepi	gooseberry	gsb	A0A182PSD0_9DIPT	EMBL-EBI reference proteomes		
Anopheles epiroticus	Aepi	eyeless/paired box 6	ey/pax6	A0A182PFT9_9DIPT	EMBL-EBI reference proteomes		
Anopheles epiroticus	Aepi	paired box 6-like	pax6-like	A0A182PFU4_9DIPT	EMBL-EBI reference proteomes		
Anopheles farauti	Afar	eyegone	eyg	A0A182Q9U1_9DIPT	EMBL-EBI reference proteomes		
Anopheles farauti	Afar	gooseberry	gsb	A0A182Q611_9DIPT	EMBL-EBI reference proteomes		
Anopheles farauti	Afar	eyeless/paired box 6	ey/pax6	A0A182QVU0_9DIPT	EMBL-EBI reference proteomes		
Anopheles farauti	Afar	paired box 6-like	pax6-like	A0A182QGD1_9DIPT	EMBL-EBI reference proteomes		
Anopheles funestus	Afun	eyegone	eyg	A0A182RFD8_ANOFN	EMBL-EBI reference proteomes		
Anopheles funestus	Afun	gooseberry	gsb	A0A182R909_ANOFN	EMBL-EBI reference proteomes		
Anopheles funestus	Afun	eyeless/paired box 6	ey/pax6	A0A182RZ39_ANOFN	EMBL-EBI reference proteomes		
Anopheles funestus	Afun	paired box 6-like	pax6-like	A0A182RZ44_ANOFN	EMBL-EBI reference proteomes		
Anopheles gambiae	Agam	eyegone	eyg	Q7PN11_ANOGA	EMBL-EBI reference proteomes		
Anopheles gambiae	Agam	gooseberry	gsb	AGAP010358	Vectorbase	Q5TUG5_ANOGA	EMBL-EBI reference proteomes
Anopheles gambiae	Agam	gooseberry-neuro	gsb-n	AGAP010359	Vectorbase	Q7PRH9_ANOGA	EMBL-EBI reference proteomes
Anopheles gambiae	Agam	eyeless/paired box 6	ey/pax6	AGAP000067	Vectorbase	Q7QEM9_ANOGA	EMBL-EBI reference proteomes
Anopheles gambiae	Agam	paired box 6-like	pax6-like	Q5TUU2_ANOGA	EMBL-EBI reference proteomes		
Anopheles maculatus	Amac	eyegone	eyg	A0A182TA71_9DIPT	EMBL-EBI reference proteomes		
Anopheles maculatus	Amac	gooseberry	gsb	A0A182SE63_9DIPT	EMBL-EBI reference proteomes		
Anopheles melas	Amela	eyegone	eyg	A0A182U1W1_9DIPT	EMBL-EBI reference proteomes		
Anopheles melas	Amela	gooseberry	gsb	A0A182TPA9_9DIPT	EMBL-EBI reference proteomes		
Anopheles melas	Amela	gooseberry-neuro	gsb-n	A0A182TY28_9DIPT	EMBL-EBI reference proteomes		
Anopheles melas	Amela	eyeless/paired box 6	ey/pax6	A0A182U339_9DIPT	EMBL-EBI reference proteomes		
Anopheles melas	Amela	paired box 6-like	pax6-like	A0A182UK21_9DIPT	EMBL-EBI reference proteomes		
Anopheles merus	Amer	eyegone	eyg	A0A182UUW9_ANOME	EMBL-EBI reference proteomes		
Anopheles merus	Amer	gooseberry	gsb	A0A182UTD8_ANOME	EMBL-EBI reference proteomes		
Anopheles merus	Amer	gooseberry-neuro	gsb-n	A0A182V289_ANOME	EMBL-EBI reference proteomes		
Anopheles merus	Amer	eyeless/paired box 6	ey/pax6	A0A182US60_ANOME	EMBL-EBI reference proteomes		
Anopheles minimus	Amin	eyegone	eyg	A0A182WF21_9DIPT	EMBL-EBI reference proteomes		
Anopheles minimus	Amin	gooseberry	gsb	A0A182W878_9DIPT	EMBL-EBI reference proteomes		
Anopheles minimus	Amin	eyeless/paired box 6	ey/pax6	A0A182VVN7_9DIPT	EMBL-EBI reference proteomes		
Anopheles minimus	Amin	paired box 6-like	pax6-like	A0A182VVN2_9DIPT	EMBL-EBI reference proteomes		
Anopheles quadriannulatus	Aqua	eyegone	eyg	A0A182WR51_ANOQN	EMBL-EBI reference proteomes		
Anopheles quadriannulatus	Aqua	gooseberry	gsb	A0A182XA89_ANOQN	EMBL-EBI reference proteomes		
Anopheles quadriannulatus	Aqua	gooseberry-neuro	gsb-n	A0A182XA88_ANOQN	EMBL-EBI reference proteomes		
Anopheles quadriannulatus	Aqua	eyeless/paired box 6	ey/pax6	A0A182XER0_ANOQN	EMBL-EBI reference proteomes		
Anopheles quadriannulatus	Aqua	paired box 6-like	pax6-like	A0A182XEQ5_ANOQN	EMBL-EBI reference proteomes		
Anopheles sinensis	Asin	eyegone	eyg	A0A084WI76_ANOSI	EMBL-EBI reference proteomes		
Anopheles sinensis	Asin	gooseberry	gsb	A0A084WC88_ANOSI	EMBL-EBI reference proteomes		
Anopheles sinensis	Asin	eyeless/paired box 6	ey/pax6	A0A084WPR6_ANOSI	EMBL-EBI reference proteomes		
Anopheles sinensis	Asin	paired box 6-like	pax6-like	A0A084WPS2_ANOSI	EMBL-EBI reference proteomes		
Anopheles stephensi	Aste	gooseberry	gsb	ASTEI06722	Vectorbase	A0A182YE36_ANOST	EMBL-EBI reference proteomes
Anopheles stephensi	Aste	gooseberry-neuro	gsb-n	ASTEI06723	Vectorbase	A0A182YE37_ANOST	EMBL-EBI reference proteomes
Anopheles stephensi	Aste	eyeless/paired box 6	ey/pax6	ASTEI06205	Vectorbase	A0A182YCL9_ANOST	EMBL-EBI reference proteomes
Anopheles stephensi	Aste	paired box 6-like	pax6-like	A0A182YCL4_ANOST	EMBL-EBI reference proteomes		
Apis melifera	Amel	gooseberry	gsb	XP_006559357.1 and XM_016916445	Genbank		
Apis melifera	Amel	gooseberry-neuro	gsb-n	XP_006559358.1 and XM_006559295	Genbank		
Apis melifera	Amel	paired	prd	XP_006563032.1 amd XM_006562969	Genbank		
Belgica Antarctica	Bant	gooseberry	gsb	IU25_01014	Ensembl Metazoa		
Belgica Antarctica	Bant	gooseberry-neuro	gsb-n	IU25_10914	Ensembl Metazoa		
Belgica Antarctica	Bant	eyeless/paired box 6	ey/pax6	IU25_08052	Ensembl Metazoa		
Belgica Antarctica	Bant	eyeless/paired box 6	ey/pax6	IU25_07260	Ensembl Metazoa		
Belgica Antarctica	Bant	Paired	prd	IU25_07661	Ensembl Metazoa		
Clogmia albipunctata	Calb	gooseberry	gsb	CAL_comp5619_c0_seq1	http://diptex.crg.es		
Clogmia albipunctata	Calb	paired	prd	CAL_comp5293_c0_seq1	http://diptex.crg.es		
Culex quinquefsciatus	Cqui	gooseberry	gsb	CPIJ006496	Vectorbase	B0WHJ6	EMBL-EBI Reference proteomes
Culex quinquefsciatus	Cqui	gooseberry-neuro	gsb-n	CPIJ019791	Vectorbase		
Culex quinquefsciatus	Cqui	gooseberry-neuro	gsb-n	CPIJ006494	Vectorbase		
Culex quinquefsciatus	Cqui	Eyeless/Paired box 6	ey/pax6	CPIJ00639	Vectorbase	B0WHG3	EMBL-EBI Reference proteomes
Culex quinquefsciatus	Cqui	paired box 6-like	pax6-like	CPIJ006390	Vectorbase	B0WHF5	EMBL-EBI Reference proteomes
Culex quinquefsciatus	Cqui	twin of eyeless	toy	B0WHG3	EMBL-EBI Reference proteomes		

Supplemental Table 1 continued

Species	Species abbreviation	Gene name	Gene abbreviation	Accession number or annotation ID	source	Alternate accession or annotation ID	Alternate source
Culicoides sonorensis	*Cson*	*gooseberry*	*gsb*	OGVF02001914.1 OGVF02001344.1	Genbank		
Culicoides sonorensis	*Cson*	*paired*	*prd*	OGVF02000011.1	Genbank		
Dermestes maculatus	*Dmac*	*gooseberry*	*gsb*	KT875128.1	Genbank		
Dermestes maculatus	*Dmac*	*gooseberry-neuro*	*gsb-n*	NM_079138.2	Genbank		
Dermestes maculatus	*Dmac*	*paired*	*prd*	KT875123.1	Genbank		
Drosophila melanogaster	*Dmel*	*eyeless/paired box 6*	*ey/pax6*	O18381.3	Genbank	Q59DQ1 and Q59DQ2	EMBL-EBI Reference proteomes
Drosophila melanogaster	*Dmel*	*eyegone*	*eyg*	Q9VTX7	*EMBL-EBI Reference proteomes*		
Drosophila melanogaster	*Dmel*	*gooseberry*	*gsb*	NM_079139.4	Genbank	P09082	EMBL-EBI Reference proteomes
Drosophila melanogaster	*Dmel*	*gooseberry-neuro*	*gsb-n*	NM_079138.2	Genbank	P09083	EMBL-EBI Reference proteomes
Drosophila melanogaster	*Dmel*	*paired*	*prd*	M14548.1	Genbank	P06601-1 and P06601-2	EMBL-EBI Reference proteomes
Drosophila melanogaster	*Dmel*	*twin of eyegone*	*toe*	M9PFA4 and Q8T0M4	*EMBL-EBI Reference proteomes*		
Drosophila melanogaster	*Dmel*	*twin of eyeless*	*toy*	Q9V490 and L0MPR7	EMBL-EBI Reference proteomes		
Lutzomyia longipalpis	*Llon*	*eyeless/paired box 6*	*ey/pax6*	LLOJ004809	Vectorbase		
Lutzomyia longipalpis	*Llon*	*paired*	*prd*	LLOJ009941	Vectorbase		
Mus musculus	*Mmus*	*pax3*	*pax3*	BC048699.1	Genbank		
Mus musculus	*Mmus*	*pax7*	*pax7*	U20792.1	Genbank		
Mus musculus	*Mmus*	*eyeless/paired box 6*	*ey/pax6*	BC036957.1	Genbank		
Musca domestica	*Mdom*	*eyeless/paired box 6*	*ey/pax6*	XM_011296630.2	Genbank		
Musca domestica	*Mdom*	*gooseberry*	*gsb*	XM_005182004	Genbank		
Musca domestica	*Mdom*	*gooseberry-neuro*	*gsb-n*	XM_005182005	Genbank		
Musca domestica	*Mdom*	*paired*	*prd*	XM_005175850	Genbank		
Phlebotomus papatasi	*Ppap*	*eyeless/paired box 6*	*ey/pax6*	PPAI009002	Vectorbase		
Phlebotomus papatasi	*Ppap*	*paired*	*prd*	PPAI006228	Vectorbase		
Tribolium castaneum	*Tcas*	*eyeless/paired box 6*	*ey/pax6*	NM_001110437.1	Genbank		
Tribolium castaneum	*Tcas*	*gooseberry*	*gsb*	XM_008199231.1	Genbank		
Tribolium castaneum	*Tcas*	*gooseberry-neuro*	*gsb-n*	XM_969092.2	Genbank		
Tribolium castaneum	*Tcas*	*paired*	*prd*	NM_001077622.1	Genbank		

Supplemental Table 2

Species	Species abbreviation	Classification and description	Gene name	Gene abbreviation	Accession number or annotation ID	source	alt accession	alt source
Acyrthosiphon pisum	*Apis*	Insecta, Hemiptera (aphid)	*paired*	*prd*	XM_008188021	Genbank		
Acyrthosiphon pisum	*Apis*	Insecta, Hemiptera (aphid)	*gooseberry*	*gsb*	XM_008185746	Genbank		
Acyrthosiphon pisum	*Apis*	Insect, Hemiptera (aphid)	*gooseberry*	*gsb*	XM_008185746	Genbank		
Anopheles stephensi	*Aste*	Diptera (mosquito)	*gooseberry*	*gsb*	ASTEI06722	Vectorbase	ASTEI06722	ENSEMBL Metazoa
Anopheles stephensi	*Aste*	Diptera (mosquito)	*gooseberry-neuro*	*gsb-n*	ASTEI06723	Vectorbase	ASTEI06723	ENSEMBL Metazoa
Anopheles stephensi	*Aste*	Diptera (mosquito)	*eyeless/paired box 6*	*ey/pax6*	ASTEI06205	Vectorbase	ASTEI06205	ENSEMBL Metazoa
Apis mellifera	*Amel*	Insecta, Hymenoptera (honeybee)	*gooseberry*	*gsb*	XM_016916445	Genbank	GB43792	ENSEMBL Metazoa
Apis mellifera	*Amel*	Insecta, Hymenoptera (honeybee)	*paired*	*prd*	XM_006562969	Genbank	GB54180	ENSEMBL Metazoa
Apis mellifera	*Amel*	Insecta, Hymenoptera (honeybee)	*gooseberry-neuro*	*gsb-n*	XM_006559295	Genbank	GB43794	ENSEMBL Metazoa
Blattella germanica	*Bger*	Insecta, Polyneoptera, Dictyoptera (cockroach)	*paired*	*prd*	Bger_05081	i5K (USDA)		
Blattella germanica	*Bger*	Insecta, Polyneoptera, Dictyoptera (cockroach)	*gooseberry-neuro*	*gsb-n*	blager_Scaffold988	i5K (USDA)		
Branchiostoma floridae	*Bflo*	Chordata (lancelet)	*pax3/7*	*pax3/7*	MF979123.1	Genbank		
Catajapyx aquilonaris	*Caqu*	Insecta, Diplura (bristletail)	*gooseberry*	*gsb*	scaffold3814	i5K (USDA)		
Clitarchus hookeri	*Choo*	Insect, Polyneoptera, Phasmatodea (stick insect)	*gooseberry*	*gsb*	NQII01000701.1	i5K (USDA)	scaffold 702	
Cupiennius salei	*Csal*	Chelicerata (spider)	*pax3/7-1, "pairberry-1"*	*pby1*	AJ744784	Genbank		
Cupiennius salei	*Csal*	Chelicerata (spider)	*pax3/7-2, "pairberry-2"*	*pby2*	AJ744785	Genbank		
Cupiennius salei	*Csal*	Chelicerata (spider)	*pax3/7-3, "pairberry-3"*	*pby3*	AJ744786	Genbank		
Danaus plexippus	*Dple*	Insecta, Lepidoptera (monarch butterfly)	*gooseberry*	*gsb*	DPOGS216048	ENSEMBL Metazoa		
Danaus plexippus	*Dple*	Insecta, Lepidoptera (monarch butterfly)	*gooseberry-neuro*	*gsb-n*	DPOGS216059	ENSEMBL Metazoa		
Daphnia magna	*Dmag*	Crustacea (Branchiopoda)	*gooseberry-1*	*gsb-1*	APZ42_020326	ENSEMBL Metazoa		
Daphnia magna	*Dmag*	Crustacea (Branchiopoda)	*gooseberry-2*	*gsb-2*	APZ42_020328	ENSEMBL Metazoa		
Daphnia magna	*Dmag*	Crustacea (Branchiopoda)	*paired*	*prd*	APZ42_015901	ENSEMBL Metazoa		
Drosophila melanogaster	*Dmel*	Diptera (fruit fly)	*paired*	*prd*	M14548.1	Genbank	prd	ENSEMBL Metazoa
Drosophila melanogaster	*Dmel*	Diptera (fruit fly)	*gooseberry*	*gsb*	NM_079139.4	Genbank	gsb-n	ENSEMBL Metazoa
Drosophila melanogaster	*Dmel*	Diptera (fruit fly)	*gooseberry-neuro*	*gsb-n*	NM_079138.2	Genbank	gsb-n	ENSEMBL Metazoa
Drosophila melanogaster	*Dmel*	Diptera (fruit fly)	*eyeless/paired box 6*	*ey*	NM_166789	Genbank		
Ephemera danica	*Edan*	Insect, Ephemeroptera (mayfly)	*gooseberry*	*gsb*	EDAN013385	i5K (USDA)		
Euperipatoides rowelli	*Erow*	Onychophora (velvet worm)	*pax3/7*	*pax3/7*	KJ957833.1	Genbank		
Eurytemora affinis	*Eaff*	Crustacea (Copepoda)	*paired*	*prd*	XM_023487636	Genbank		
Eurytemora affinis	*Eaff*	Crustacea (Copepoda)	*gooseberry-1*	*gsb1*	XM_023470322	Genbank		
Eurytemora affinis	*Eaff*	Crustacea (Copepoda)	*gooseberry-3*	*gsb3*	XM_023470308	Genbank		
Eurytemora affinis	*Eaff*	Crustacea (Copepoda)	*gooseberry-2*	*gsb2*	XM_023470312	Genbank		
Folsomia candida	*Fcan*	Hexapod, Collembola	*paired-2*	*prd2*	Fcan01_12273	ENSEMBL Metazoa	XM_022098541	Genbank
Folsomia candida	*Fcan*	Hexapod, Collembola	*paired-1*	*prd1*	Fcan01_12272	ENSEMBL Metazoa	XM_022098569	Genbank
Folsomia candida	*Fcan*	Hexapod, Collembola	*gooseberry*	*gsb*	Fcan01_07645	ENSEMBL Metazoa	XM_022093744	Genbank
Glomeris marginata	*Gmar*	Myriapoda (millipede)	*pax3/7-2, "pairberry-2"*	*pby2*	FR715038.1	Genbank		
Glomeris marginata	*Gmar*	Myriapoda (millipede)	*pax3/7-1, "pairberry-1"*	*pby1*	FR715037.1	Genbank		
Hyalella azteca	*Hazt*	Crustacea (Amphipoda)	*paired*	*prd*	XM_018162991	Genbank		
Ixodes scapularis	*Isca*	Chelicerata (tick)	*pax3/7*	*pax3/7*	XM_029968122	genbank		
Manduca sexta	*Msex*	Insecta, Lepidoptera (Carolina sphinx moth)	*gooseberry*	*gsb*	XM_030184640	Genbank		
Manduca sexta	*Msex*	Insecta, Lepidoptera (Carolina sphinx moth)	*gooseberry-neuro*	*gsb-n*	XM_030184557	Genbank		
Mus musculus	*Mmus*	Vertebrata (mouse)	*pax7*	*pax7*	U20792.1	Genbank		
Mus musculus	*Mmus*	Vertebrata (mouse)	*pax3*	*pax3*	BC048699.1	Genbank		
Mus musculus	*Mmus*	Vertebrata (mouse)	*eyeless/paired box 6*	*ey/pax6*	BC036957.1	Genbank		
Musca domestica	*Mdom*	Insecta, Diptera (house fly)	*paired*	*prd*	XM_005175850	Genbank		
Musca domestica	*Mdom*	Insecta, Diptera (house fly)	*gooseberry*	*gsb*	XM_005182004	Genbank		
Musca domestica	*Mdom*	Insecta, Diptera (house fly)	*gooseberry-neuro*	*gsb-n*	XM_005182005	Genbank		
Nasonia vitripennis	*Nvit*	Insecta, Hymenoptera (parasitoid wasp)	*paired*	*prd*	GQ301535	Genbank		
Oncopeltus fasciatus	*Ofas*	Insecta, Hemiptera (milkweed bug)	*paired*	*prd*	MT596737	Genbank		
Oncopeltus fasciatus	*Ofas*	Insecta, Hemiptera (milkweed bug)	*gooseberry*	*gsb*	MT596738	Genbank		
Orchesella cincta	*Ocin*	Hexapod, Collembola	*paired*	*prd*	Ocin01_02469	ENSEMBL Metazoa		
Orchesella cincta	*Ocin*	Hexapod, Collembola	*gooseberry*	*gsb*	Ocin01_14031	ENSEMBL Metazoa		
Parhyale hawaiensis	*Phaw*	crustacea (amphipod)	*pax3/7*	*pax3/7*	HM347085.1	Genbank		
Pediculus humanus	*Phum*	Insect, Paraneoptera, Psocodea (louse)	*paired*	*prd*	PHUM134580	ENSEMBL Metazoa		
Pediculus humanus	*Phum*	Insect, Paraneoptera, Psocodea (louse)	*pax1/9*	*pax1/9*	PHUM515110	ENSEMBL Metazoa		
Pediculus humanus	*Phum*	Insect, Paraneoptera, Psocodea (louse)	*gooseberry*	*gsb*	PHUM375940	ENSEMBL Metazoa		
Schistocerca americana	*Same*	Insect, Polyneoptera, Orthoptera (grasshopper)	*gooseberry*	*gsb*	AY040535.1	Genbank		
Schistocerca americana	*Same*	Insect, Polyneoptera, Orthoptera (grasshopper)	*gooseberry-neuro*	*gsb-n*	AY040536.1	Genbank		
Solenopsis invicta	*Sinv*	Insecta, Hymenoptera (fire ant)	*paired*	*prd*	LOC105196789	ENSEMBL Metazoa	XM_011162899.1	Genbank
Solenopsis invicta	*Sinv*	Insecta, Hymenoptera (fire ant)	*gooseberry*	*gsb*	LOC105207407	ENSEMBL Metazoa	XM_011176850.1	Genbank
Solenopsis invicta	*Sinv*	Insecta, Hymenoptera (fire ant)	*gooseberry-neuro*	*gsb-n*	LOC105207408	ENSEMBL Metazoa	XM_011176851.1	Genbank
Solenopsis invicta	*Sinv*	Insecta, Hymenoptera (fire ant)	*pax1/9*	*pax1/9*	LOC105194388	ENSEMBL Metazoa	XM_011159271.1	Genbank
Strigamia maritima	*Smar*	Myriapoda (centipede)	*pax1/9*	*pax1/9*	SMAR007866	ENSEMBL Metazoa		
Strigamia maritima	*Smar*	Myriapoda (centipede)	*pax3/7-1, "pairberry-1"*	*pby1*	SMAR015380 *	ENSEMBL Metazoa		
Tetranychus urticae	*Turt*	Chelicerata (mite)	*pax3/7-2*	*pax3/7-2*	XM_015928659	Genbank		
Tetranychus urticae	*Turt*	Chelicerata (mite)	*"pax3/7"*	*pax3/7*	XM_015936900	Genbank	AY148194.1	Genbank
Tribolium casteneum	*Tcas*	Insecta, Coleoptera (flour beetle)	*gooseberry-neuro*	*gsb-n*	XM_969092	Genbank	TC005342	ENSEMBL Metazoa
Tribolium casteneum	*Tcas*	Insecta, Coleoptera (flour beetle)	*gooseberry*	*gsb*	XM_008199231	Genbank	TC006788	ENSEMBL Metazoa
Tribolium casteneum	*Tcas*	Insecata, Coleoptera (flour beetle)	*paired*	*prd*	NM_001077622	Genbank	TC015804	ENSEMBL Metazoa
Zootermopsis nevadensis	*Znav*	Insecta, Polyneoptera, Dictyoptera (termite)	*paired*	*prd*	XM_022069086	Genbank		
Zootermopsis nevadensis	*Znav*	Insecta, Polyneoptera, Dictyoptera (termite)	*gooseberry*	*gsb*	XM_022086663	Genbank		
Zootermopsis nevadensis	*Znav*	Insecta, Polyneoptera, Dictyoptera (termite)	*gooseberry-neuro*	*gsb-n*	XM_022086661	Genbank		

*also included non-annotated genomic hit adjacent to predicted gene to complete protein

Supplemental Table 3

gsb loci

	gene 5 5'	gene 4 5'	gene 3 5'	gene 2 5'	gene 1 5'	gsb	gene 1 3'	gene 2 3'	gene 3 3'	gene 4 3'	gene 5 3'
Aedes aegypti Chr3	AAEL011634 no orthology	AAEL011633 no orthology	AAEL014873 osi21	AAEL013882 lov	AAEL012337 gol	AAEL02370 gsb	AAEL021574 gsb-n	AAEL012649 no orthology	AAEL012641 no orthology	AAEL015252 no orthology	AAEL01523 no orthology
Musca domestica scaffold 18963				MDOA009101 lov	MDOA004560 gol	MDOA008527 gsb	MDOA002110 gsb-n	MDOA009959 Nplp1	MDOA008373 uzip	MDOA013000 CG10257	MDOA005070 no orthology
Apis melifera chr 14	GB43788 TC005541	GB43789 TTLL4B	GB43790 TTLL4A	GB43791 Hesr	GB43773 no orthology	GB43792 gsb	GB43793 no orthology	GB43794 gsb-n	GB43772 no orthology	GB43771 no orthology	GB43771 WDR79
Tribolium castaneum LG8	TC005345 wls	TC005344 GB49311	TC005843 no orthology	TC006787 no orthology	TC005342 gsb-n	TC006788 gsb	TC034852 no orthology	TC005341 mod	TC006790 no orthology	TC034192 no orthology	TC006791 no orthology
Anopheles stephensi scaffold 00043	AsteI06717 Osi21	AsteI06718 lov	AsteI06719 lov	AsteI06720 gol	AsteI06721 CPIJ006498	AsteI06722 gsb	AsteI06723 gsb-n	AsteI06724 no orthology	AsteI06725 CPIJ006495	AsteI06726 CPIJ006495	AsteI06727 Nplp1
Culex quinquefasciatus Supercontig 3.125	CPIJ006501 Osi21	CPIJ006500 lov	CPIJ006499 lov	CPIJ006498 no orthology	CPIJ006497 gol	CPIJ006496 gsb	CPIJ006495 AsteI06725	CPIJ006494 gsb-n	CPIJ006493 no orthology		
Drosophila melanogaster Chr 2R	CG34038	CG43106	CG30430	lov	gol	gsb	gsb-n	Nplp1	uzip	zip	CG3829

prd loci

	gene 5 5'	gene 4 5'	gene 3 5'	gene 2 5'	gene 1 5'	prd	gene 1 3'	gene 2 3'	gene 3 3'	gene 4 3'	gene 5 3'
Musca domestica scaffold 1821	MDOA002413 CG15168	MDOA008869 no orthology	MDOA015497 no orthology	MDOA001652 no orthology	MDOA005888 no orthology	MDOA004461 prd	MDOA007305 no orthology	MDOA003617 CG6712	MDOA016638 fabp	MDOA014307 Megf8	MDOA015061 Pgk
Apis melifera chr 14	GB54124 dtr	GB54181 GB13F	GB54125 no orthology	GB54126 no orthology	GB54127 sei	GB54180 prd	GB54128 no orthology	GB54179 TC033801	GB54178 no orthology	GB54129 Lsm12a	GB54177 CG10185
Tribolium castaneum LG6	TC0157987 no orthology	TC015801 no orthology	TC014911 POLDIP2	TC015802 CG5281	TC015803 no orthology	TC015804 prd	TC014910 GB48266	TC015805 GB41660	TC015806 no orthology	TC015807 no orthology	TC014909 CG9701
Drosophila melanogaster Chr 2L	Rp17-like	CG34164	Rab3-GAP	firl	CG14947	prd	pex19	CG6712	Mt2	Ced-12	Patsas
Phlebotomus papatasi scaffold 4	PPAI006223 no orthology	PPAI006224 Ets21c	PPAI006225 CG5676	PPAI006226 Rab3-GAP	PPAI006227 firl	PPAI006228 prd	PPAI006229 no orthology	PPAI006230 CG6295	PPAI006231 CG7997	PPAI006232 CG9967	PPAI006233 Rab5
Belgica antarctica Supercontig JPYR01000363		IU25_076577 CG9864	IU25_07658 Ate1	IU25_07659 CG34039	IU25_07660 no orthology	IU25_07661 prd	IU25_07662 no orthology	IU25_07663 no orthology			
Culicoides sonorensis scaffold 1914	CSON001321 no orthology	CSON001322 CG9773	CSON001323 blanks	CSON001324 Got2	CSON001325 no orthology	CSON001326 prd	CSON001327 mura	CSON001329 CCT7	CSON001330 CG11954	CSON001332 MDOA011652	CSON001333 Bant IU25_00534

Supplemental Table 4

Species	Gene name	Annotation Symbol (FlyBase)	Probe template type	cDNA clone ID	PCR primer (5')	PCR primer (3')
Drosophila melanogaster	*engrailed*	CG9015	plasmid	LD16125		
Drosophila melanogaster	*evenskipped*	CG2328	plasmid	MIP30861		
Drosophila melanogaster	*ftz-f1*	CG4059	PCR	N/A	ATGTACCTATGCTGGCGGAG	GACAGGAAGTTGCTGGTGGA
Drosophila melanogaster	*fushi tarazu*	CG2047	plasmid	IP01266		
Drosophila melanogaster	*gooseberry*	CG3388	PCR	N/A	CCAGCAGGTGCCTAGCTTTG	AGACGATCCCTGCTTGTTCC
Drosophila melanogaster	*hairy*	CG6494	plasmid	RE40955		
Drosophila melanogaster	*oddpaired*	CG1133	PCR	N/A	CATGGCAATGTGGACGAGAAGAG	AACCATTTCGGACACCATCACC
Drosophila melanogaster	*oddskipped*	CG3851	plasmid	RE48009		
Drosophila melanogaster	*paired*	CG6716	plasmid	GH22686		
Drosophila melanogaster	*runt*	CG1849	plasmid	GH02614		
Drosophila melanogaster	*sloppy paired-1*	CG16738	PCR	N/A	AGAGCTAGCATCTCAAGAGC	CATACAGCTACAACGCCCTC
Drosophila melanogaster	*wingless*	CG4489	plasmid	RE02607		
		(Vectorbase)				
Anopheles stephensi	*engrailed*	ASTE006297	PCR	N/A	CTGGAAGACCGCTGTAGTCC	GCCTTCGTTTGCGATTCGAT
Anopheles stephensi	*evenskipped*	ASTEI09848	PCR	N/A	GACAAACGGCAACGGATAGC	CGCTTCGGACTTGTAAGGCT
Anopheles stephensi	*ftz-f1*	ASTEI02250	PCR	N/A	GGACGATCAGATGAAGCTGC	GCATGCAGCATCTCCATCAG
Anopheles stephensi	*fushi tarazu*	ASTEI06924	PCR	N/A	ACTATGCGCCATTTGGACAG	CCGTGTGATGAGATTGCACT
Anopheles stephensi	*gooseberry*	ASTEI06722	PCR	N/A	GAGCTCCCAGCAAATGGTGG	CAGTGCAGTCGGCTCATCTC
Anopheles stephensi	*hairy*	ASTEI10025	PCR	N/A	ACATCCTGGAGATGACGGTG	GTCCTCCTGCTTCATCTCGT
Anopheles stephensi	*oddpaired*	ASTEI05790	PCR	N/A	GGAGCAGAGCAGTGAAATCG	GATGGTGGTGGTGATGATGG
Anopheles stephensi	*oddskipped*	ASTEI01737	PCR	N/A	TGTGAAATCGGTCCCATCATCG	GGTCCACCGTGATGATGGCC
Anopheles stephensi	*runt*	ASTEI07770	PCR	N/A	GGACATCCGGGTGCATTTAATC	GAGCGAAAGGCGGCTATGTC
Anopheles stephensi	*sloppy paired-1*	ASTEI06173	PCR	N/A	GGGTAACTACTGGATGCTGG	CATGCTGCTGCAGAAGATGC
Anopheles stephensi	*wingless*	ASTE003105	PCR	N/A	CAGCAGTAACGCACAGTGTG	GGCACGTGTGGATGATCTTT

Supplemental Table 5

primer name	primer sequence	purpose
AsteGsb_FLHDR_F	AAAGGTGTCCGCAACTTCAGG	construction of HDR donor plasmid
AsteGsb_leftarm3xP3_F	CGAGTGTGGTTCTGGGTTGTGGTTCCCACAATGGTTAATTC	construction of HDR donor plasmid
AsteGsb_leftarm3xP3_R	GAATTAACCATTGTGGGAACCACAACCCAGAACCACACTCG	construction of HDR donor plasmid
AsteGsb_3xP3rightarm_F	TCCAAACTCATCAATGTATCTTAGAGATGGCGTCCTCCGGAGT	construction of HDR donor plasmid
AsteGsb_3xP3rightarm_R	ACTCCGGAGGACGCCATCTCTAAGATACATTGATGAGTTTGGA	construction of HDR donor plasmid
AsteGsb_FLHDR_R	GACGGTGCATGAATCGAAGC	construction of HDR donor plasmid
gRNArev	AAAAGCACCGACTCGGTGCC	production of PCR template for gRNA synthesis
AsteGsbgRNA1	TTAATACGACTCACTATAGGATTGTCACACAGCACAACCCGTTTTAGAGCTAGAAATAG	production of PCR template for gRNA synthesis
AsteGsbgRNA2	TTAATACGACTCACTATAGGACTGCGCATCGTCGAGATGGGTTTTAGAGCTAGAAATAG	production of PCR template for gRNA synthesis
AsteGsbCRISPRscreen1	ATCAAAGACCGGCGAGGAAG	initial PCR screening of potential positive transformants by inejction facility
AsteGsbCRISPRscreen2	CCTTAATGAGCTTGTCCCGGA	initial PCR screening of potential positive transformants by inejction facility
AsteGsb HDR confirm 1	AAGACGGAATGCCGCGGAATG	amplification and sequencing of gsb locus after CRISPR HDR
AsteGsb HDR confirm 2	GGACCGAAGTTTGTGGGATA	amplification and sequencing of gsb locus after CRISPR HDR
AsteGsb HDR confirm 3	GGAGCCTCTGCTTCTGTTTC	amplification and sequencing of gsb locus after CRISPR HDR

Supplementary References

1. Green, J. & Akam, M. Evolution of the pair rule gene network: Insights from a centipede. *Dev. Biol.* **382**, 235–245 (2013).
2. Misof, B. *et al.* Phylogenomics resolves the timing and pattern of insect evolution. *Science* **346**, 763–767 (2014).
3. Wiegmann, B. M. *et al.* Episodic radiations in the fly tree of life. *Proc. Natl. Acad. Sci. U. S. A.* **108**, 5690–5695 (2011).
