# Supplementary material for: Regulatory gene function handoff allows essential gene loss in mosquitoes
Source: Commun Biol. 2020 Sep 30;3:540. doi: 10.1038/s42003-020-01203-w (PMC7528073; doi:10.1038/s42003-020-01203-w)
Supplement: Supplementary file 3 — Reporting Summary [file 42003_2020_1203_MOESM3_ESM.pdf]

nature research

Corresponding author(s): Leslie Pick

Last updated by author(s): Jul 20, 2020

Reporting Summary

Nature Research wishes to improve the reproducibility of the work that we publish. This form provides structure for consistency and transparency in reporting. For further information on Nature Research policies, see our Editorial Policies and the Editorial Policy Checklist.

Statistics

For all statistical analyses, confirm that the following items are present in the figure legend, table legend, main text, or Methods section.

n/a	Confirmed	
☐	☒	The exact sample size (*n*) for each experimental group/condition, given as a discrete number and unit of measurement
☐	☒	A statement on whether measurements were taken from distinct samples or whether the same sample was measured repeatedly
☐	☒	The statistical test(s) used AND whether they are one- or two-sided<br>*Only common tests should be described solely by name; describe more complex techniques in the Methods section.*
☐	☒	A description of all covariates tested
☒	☐	A description of any assumptions or corrections, such as tests of normality and adjustment for multiple comparisons
☐	☒	A full description of the statistical parameters including central tendency (e.g. means) or other basic estimates (e.g. regression coefficient) AND variation (e.g. standard deviation) or associated estimates of uncertainty (e.g. confidence intervals)
☐	☒	For null hypothesis testing, the test statistic (e.g. *F*, *t*, *r*) with confidence intervals, effect sizes, degrees of freedom and *P* value noted<br>*Give P values as exact values whenever suitable.*
☒	☐	For Bayesian analysis, information on the choice of priors and Markov chain Monte Carlo settings
☒	☐	For hierarchical and complex designs, identification of the appropriate level for tests and full reporting of outcomes
☐	☒	Estimates of effect sizes (e.g. Cohen's *d*, Pearson's *r*), indicating how they were calculated

Our web collection on statistics for biologists contains articles on many of the points above.

Software and code

Policy information about availability of computer code

Data collection	EMBL-EBI HmmerWeb version 2.33.0, https://www.ebi.ac.uk/Tools/hmmer/search/hmmsearch
Data analysis	TOPALI v2.5 build 13.04.03 (www.topali.org)<br>NCBI conserved domains batch web search tool (https://www.ncbi.nlm.nih.gov/Structure/bwrpsb/bwrpsb.cgi<br>Genomicus Metazoa (web-code version: 2014-07-06, database version: 30.01, https://www.genomicus.biologie.ens.fr/genomicus-metazoa-30.01/cgi-bin/search.pl

For manuscripts utilizing custom algorithms or software that are central to the research but not yet described in published literature, software must be made available to editors and reviewers. We strongly encourage code deposition in a community repository (e.g. GitHub). See the Nature Research guidelines for submitting code & software for further information.

Data

Policy information about availability of data

All manuscripts must include a data availability statement. This statement should provide the following information, where applicable:

- Accession codes, unique identifiers, or web links for publicly available datasets
- A list of figures that have associated raw data
- A description of any restrictions on data availability

No datasets were generated or analyzed during this study.

Field-specific reporting

Please select the one below that is the best fit for your research. If you are not sure, read the appropriate sections before making your selection.

☒ Life sciences ☐ Behavioural & social sciences ☐ Ecological, evolutionary & environmental sciences

For a reference copy of the document with all sections, see nature.com/documents/nr-reporting-summary-flat.pdf

Life sciences study design

All studies must disclose on these points even when the disclosure is negative.

Sample size	No sample size calculation was performed in advance. All embryos obtained from each timed collection were examined.
Data exclusions	No data were excluded.
Replication	Experiments on transgenic embryo samples were repeated multiple times by setting up equivalent experimental and control crosses with subsequent generations of the same CRISPR-induced line.
Randomization	This is not relevant to our study. Samples were not random as they each contained embryos from a pool of sibling insects. Experimental groups were carefully compared to control groups, also composed of sibling insects from the same rearing pan as the experimental group. Both crosses in a replicate experiment were constructed from the same pool of insects to control for number of outcrosses to wild type and overall genetic background, in addition to exact rearing conditions, which affect fecundity.
Blinding	Sequence comparisons (for Fig. 1 and 2 and supplementary figures) are not possible to carry out in a blinded fashion. For expression pattern analysis (Figs. 3 and 4), in situ hybridization was done by one individual (CT) and interpreted by another (AMCJ). For Figure 5, .transgenic embryo collections had to be performed in a BSL-2 facility by an approved user (AMCJ), which limited our ability to blind these experiments.

Reporting for specific materials, systems and methods

We require information from authors about some types of materials, experimental systems and methods used in many studies. Here, indicate whether each material, system or method listed is relevant to your study. If you are not sure if a list item applies to your research, read the appropriate section before selecting a response.

Materials & experimental systems

n/a	Involved in the study	
☐	☒	Antibodies
☒	☐	Eukaryotic cell lines
☒	☐	Palaeontology and archaeology
☐	☒	Animals and other organisms
☒	☐	Human research participants
☒	☐	Clinical data
☒	☐	Dual use research of concern

Methods

n/a	Involved in the study	
☒	☐	ChIP-seq
☒	☐	Flow cytometry
☒	☐	MRI-based neuroimaging

Antibodies

Antibodies used	Anti-Engrailed, Developmental Studies Hybridoma Bank, clone/catalog number 4D9, multiple lots used
Validation	Hybridoma bank website indicated Positive Tested Species Reactivity for a wide variety of arthropods at the outset of our study. Additionally, others used this same antibody successfully for another mosquito species in the same genus, Anopheles gambiae (Yoder, J.H., Carroll, S.B., 2006. The evolution of abdominal reduction and the recent origin of distinct Abdominal-B transcript classes in Diptera. Evol. Dev. 8, 241–251. https://doi.org/10.1111/j.1525-142X.2006.00095.x)

Animals and other organisms

Policy information about studies involving animals; ARRIVE guidelines recommended for reporting animal research

Laboratory animals	Anopheles stephensi, India strain
Wild animals	The study did not involve wild animals
Field-collected samples	The study did not involve animals collected from the field

Ethics oversight	Transgenic mosquitoes were housed, contained, and disposed of in accordance with protocols approved by the University of Maryland's Institutional Biosafety Committee.

Note that full information on the approval of the study protocol must also be provided in the manuscript.
